# Impact of HIV on the incidence of pre-eclampsia

**Published:** 2013-03

**Authors:** J Moodley

**Affiliations:** Department of Obstetrics and Gynaecology; Women’s Health and HIV Research Group, Department of Obstetrics and Gynaecology, University of KwaZulu-Natal, Durban, South Africa

Pre-eclampsia, a condition unique to human pregnancy, is defined as new-onset hypertension (BP ≥ 140/90 mmHg) in the second half of pregnancy, associated with significant proteinuria (≥ 30 mgms). The aetiology of this condition remains elusive but recent findings suggest that pre-eclampsia is a two-stage disorder. The first stage is thought to be due to failure of the spiral arterioles in the placental bed to undergo vascular remodelling into wide-bore channels. This vascular maladaptation of the placental bed results in a marked reduction in blood flow to the placenta and sets the scene for the second stage.

Reduction in blood flow to the placenta induces cellular hypoxia, which results in the release of trophoblastic debris, necrotic tissue and a variety of anti-angiogenic circulating factors such as soluble fms-like tyrosine kinase 1 and soluble endoglin. It is believed that these excessive anti-angiogenic factors bind with pro-angiogenic factors (vascular endothelial factor and placental growth factors), inhibiting their biological activities and subsequently resulting in widespread endothelial damage and the clinical disorder of pre-eclampsia.^1^

The current view of the pathophysiology of pre-eclampsia as described above is that this pregnancy disorder is a multi-organ endothelial disorder. Therefore it is important to recognise that although hypertension and proteinuria are the dominant clinical signs, pre-eclampsia may present with signs of isolated thrombocytopenia, liver enzyme abnormalities, intra-uterine foetal growth restriction or seizures. The exact cause however remains unknown and management is based on delaying delivery long enough for the foetus to mature, and expediting delivery of the placenta to avoid significant maternal and neonatal morbidity and mortality.^2^

However, what is generally not recognised is that hypertension may get worse following delivery or that women may present with hypertension for the first time in the immediate postpartum period (usually the first 72 hours following delivery). This is thought to reflect mobilisation of fluid accumulated in the extravascular space following delivery.

Minimal rises in blood pressure occur in normal pregnancies but more than one-third of pre-eclamptics have sustained high blood pressures in the puerperium and they may also develop pulmonary oedema. The clinical implications of this is that close monitoring of blood pressure levels must continue following delivery and it may be safer to keep all pre-eclamptics in hospital for at least three days to detect any early signs of complications and take timeous measures to prevent maternal morbidity and mortality.^3^

The initiator of the vascular maladaptation is not known but it is believed that immunological abnormalities may be involved. Similarly, HIV is an immune-dysfunction disorder and in the initial stages of this infection, when few symptoms are present, there may be a slight depression in CD_4_ T cells.^4^ It is plausible that the impaired immunity associated with HIV could lower the risk of pre-eclampsia. The current data on this matter however, are conflicting.^5^,^6^

Kalumba *et al*. took a different approach from earlier studies to establish whether HIV infection had a protective effect on the incidence of pre-eclampsia.^7^ These authors performed a retrospective case–control study by comparing HIV rates in pre-eclamptics and normotensive healthy women. Previous studies have just compared the rate of pre-eclampsia between uninfected and HIV-infected pregnant women.^5^,^8^,^9^ Kalumba *et al*. found a lower rate of HIV infection in pre-eclamptics in comparison to a control group.^7^

This study suggests that the rates of pre-eclampsia are lower in HIV-positive pregnant women. Because this study was retrospective and CD_4_ counts were not available for a large number of the study patients, there is a need for a prospective study involving large numbers of patients to confirm the findings of Kalumba *et al*.^7^
